# Increased masticatory activity and quality of life in elderly persons with dementia-a longitudinal matched cluster randomized single-blind multicenter intervention study

**DOI:** 10.1186/1471-2377-13-26

**Published:** 2013-03-16

**Authors:** Roxane Anthea Francesca Weijenberg, Frank Lobbezoo, Dirk Lucas Knol, Jori Tomassen, Erik Johan Anton Scherder

**Affiliations:** 1 Department of Clinical Neuropsychology, VU University Amsterdam, Van der Boechorststraat 1, Amsterdam, 1081 BT, The Netherlands; 2 Department of Oral Kinesiology, Academic Centre for Dentistry Amsterdam (ACTA), MOVE Research Institute Amsterdam, University of Amsterdam and VU University Amsterdam, Gustav Mahlerlaan 3004, Amsterdam, 1081 LA, The Netherlands; 3 Department of Epidemiology and Biostatistics and the EMGO Institute for Health and Care Research, VU University Medical Center, Amsterdam, The Netherlands

**Keywords:** Intervention, Protocol, Dementia, Quality of life, Oral health care, Mastication, Food consistency, Cognition

## Abstract

**Background:**

Worldwide, millions of people are suffering from dementia and this number is rising. An index of quality of life (QoL) can describe the impact a disease or treatment has on a person’s wellbeing. QoL comprises many variables, including physical health and function, and mental health and function. QoL is related to masticatory ability and physical activity. Animal studies show that disruption of mastication due to loss of teeth or a soft diet leads to memory loss and learning problems. Since these are common complaints in dementia, it is hypothesized that improvement of masticatory function and normalization of diet consistency can increase QoL in elderly persons suffering from dementia. Therefore, the goal of the present study is to examine whether an increase in masticatory activity, achieved by increased food consistency and enhancement of masticatory function through improved oral health care has a positive effect on QoL, including cognition, mood, activities of daily living (ADL), and circadian rhythm in elderly persons with dementia.

**Methods and design:**

The described study is a prospective longitudinal matched cluster randomized single-blind multicenter study. Participants are elderly persons living in the Netherlands, suffering from dementia and receiving psychogeriatric care. An intervention group will receive improved oral health care and a diet of increased consistency. A control group receives care as usual. Participants will be assessed four times; outcome variables besides QoL are cognition, mood, independence, rest-activity rhythm, blood pressure, and masticatory function.

**Discussion:**

This research protocol investigates the effect of an intervention executed by daily caregivers. The intervention will increase masticatory activity, which is achieved by three different actions, (providing oral health care, increasing food consistency, or a combination of both). There is a certain amount of variety in the nature of the interventions due to local differences in nursing homes. This might be a scientific weakness in the study design; however, a practical implementation of any findings will be subject to the same factors, making this study design clinically relevant.

**Trial registration:**

NTR1561

## Background

Dementia is an umbrella term for a heterogeneous group of neurodegenerative disorders, characterized by functionally disabling, progressive cognitive deterioration [1]. The most prevalent subtypes of dementia are Alzheimer’s disease (AD), frontotemporal dementia (FTD), dementia with Lewy bodies (DLB) and vascular dementia (VaD) [1]. These dementias can present differently; some domains that are affected in one type of dementia are preserved in others [2]. The diagnosis ‘dementia’ should therefore always be based on deficits in more than one cognitive domain [2,3]. Worldwide, almost 36 million people are suffering from dementia and this number is expected to rise to 115.4 million in 2050 [4]. This rise is mainly due to an increase in population of persons aged 60 and over [5], as aging is one of the main risk factors for dementia [6]. Genetic factors play a role as well; the apolipoprotein E (ApoE) ε4 allele is a risk factor for several types of dementia such as AD [7-10]; especially when occurring along with depression [11]. Other risk factors for dementia are female gender, illiteracy/low education, head trauma, lower socioeconomic status (SES) [6], diabetes, depression [7], vascular disorders (e.g., hypertension), environmental stress, and an inactive life style [12].

Quality of life (QoL) is a term used to describe a person’s wellbeing and more specifically, the impact a certain disease or treatment for this disease has on a person’s life [13]. QoL comprises many variables, including physical health (e.g., absence of pain and nausea), physical function (e.g., being independent in the activities of daily living (ADL)), mental health (e.g., absence of fear, agitation and mood disorders), and mental function (e.g., good cognitive function). Having a meaningful pastime and social interactions is another component that is typically included in QoL indexes [14,15]. Using questionnaires to interview caregivers such as relatives or nurses can provide some information regarding a person’s QoL. However, since the observation by such proxies can be different than the patient observation and is influenced by the mood and cognition of the caregiver him/herself [13], using additional methods to assess QoL is preferred. For example, sleep disorders influence a person’s QoL [16], and the sleep pattern and circadian rhythm can easily and objectively be tracked with actigraphy. Similarly, cognition can be measured using neuropsychological tasks. Loss of mental function is one of the more noticeable symptoms of dementia [17], and the diagnosis ‘dementia’ is usually based on cognitive testing [18]. A thorough cognitive screening should therefore also be included when studying QoL in dementia.

As there is no cure for dementia, interventions are aimed at improving the clinical consequences. For example, interventions targeting life style factors, such as increasing physical activity, have been found to improve cognitive measures in healthy elderly [19]. Physical activity interventions targeting elderly persons suffering from dementia may improve cognitive function [20], mood, and QoL [21].

Besides exercise interventions, improvement of, or supplementing the diet can positively influence cognition [22] or reduce the risk of developing cognitive impairments [23]. A ‘good’ diet would comprise fruits, nuts, and vegetables [23]; foods that are typically harder to chew. These foods become more difficult to eat for aging persons, most notably when tooth loss is present [24]. Loss of masticatory function is also associated with increased disability and mortality [25]. Being able to chew properly, therefore, is of utmost importance for elderly persons to maintain a healthy diet and preserve cognition. This is especially the case when they are at risk for, or suffering from, dementia. Unfortunately, many older persons living in a nursing home are completely edentulous [26], and if they have remaining teeth, they are often in need of dental care [27,28]. Offering both oral health care and an improved diet to elderly persons with dementia will most likely improve their health situation and thus, their QoL.

In sum, QoL is a very relevant outcome variable when assessing the results of interventions aimed at elderly persons suffering from dementia. Related mental and physical outcomes such as cognition and independence should be assessed as well to study the effects comprehensively. The study described in this protocol investigates the effect of an intervention aimed at increasing masticatory activity through improving oral health care and increased food consistency, on QoL, including cognition, mood, independence, rest-activity rhythm, and blood pressure in older people with dementia, receiving institutionalized care.

## Methods and design

### Study design

The study has a prospective longitudinal matched cluster randomized single-blind multicenter design. Assignment to either the intervention group or the control group will take place through matched cluster randomization, on a care unit level (i.e. not on an individual level). Participants in the study will be followed for 24 weeks and will be assessed at regular intervals, viz., at baseline, six weeks, twelve weeks, and 24 weeks after baseline. A care organization enrolls two care units, in order to provide matching groups within a single care setting. One group will be a control group and receive care as usual; the other group will be an intervention group. Participants are blind to the intervention. Since receiving training and altering the daily nursing care is part of the intervention, care-givers such as the nursing staff cannot be blind for the intervention. The examiners will be blinded.

### Participants

Participants are elderly persons, aged 65 years and older, diagnosed with dementia and receiving professional psychogeriatric care in a Dutch nursing home or daycare facility. If possible, the subtype of the dementia disorder (e.g., Alzheimer’s disease, vascular dementia, dementia with Lewy bodies, or frontotemporal dementia) will be noted. Exclusion criteria are: a history of psychiatric disorders, including depression, (history of) alcoholism, cerebral traumata, hydrocephalus, and brain tumors. This information will be obtained from the medical records. Another exclusion criteria will be a score on the Mini Mental State Examination (MMSE [29]: a short instrument which assesses global cognition, awareness, and memory), of 25 or higher at baseline. Such a score is contraindicative of dementia [30].

Participants are recruited through psychogeriatric care organizations. After a care organization agrees to participate in the study, all clients and their legal representatives are contacted and invited to participate in the study. They are thoroughly informed about the study, upon which written consent is obtained. Participation is always voluntary.

### Sample size

To calculate the number of participants needed, a power analysis is performed. The primary outcome variable (QoL) is used to calculate the power and a linear time effect is assumed for both intervention and control conditions. A difference on total score of 10 points after 24 weeks is considered clinically relevant. This equals to a difference of 2.5 points per 6 weeks. After running simulations with a known total variance of 300 and a mean score of 80 (pilot data), a sample size of 200 participants (spread over 10 care organizations and 10 participants per care unit) is needed. Allowing for 10% dropout, this means (200 × 10/9 =) 224 participants need to be included in the study.

### Covariates

Participants’ characteristics that might be of influence are taken into account. These covariates are age, gender, educational level, and ApoE4 status.

The highest educational level is classified by the Verhage scale [31]; a seven point rating scale. A score of 1 equals a level of less than six years of elementary school; score 2 stands for completed elementary school; a score of 3 indicates more than six years of elementary schools and less than three years of secondary education; score 4 is indicative of elementary school and three years secondary education; score 5 stands for elementary school and four years of secondary education; score 6 represents pre-university education and/or higher vocational education; and a score of 7 stands for a finished training at a university or technical college.

Genetic susceptibility for dementia as indicated by ApoE4 genotype, is assessed by collecting buccal cells. Two Catch-all TM collection swabs (Epicentre, Madison, Wisconsin, USA) per participant are used.

### Confounders

Possible confounding variables are comorbid disorders and medication usage.

Comorbid disorders such as diabetes and depression are taken into account. The diagnosis is retrieved from the medical status and classified according to the Dutch translation of the Long-Term Care Facility Resident Assessment Instrument (RAI) [32]. There are eight categories; endocrine/metabolic/nutritional; cardiovascular; locomotor system; neurology; sensory; psychiatric/mood; respiratory; other (e.g., allergies, anemia, cancer, and renal failure).

Medication usage is tracked by checking the current medical list provided by the local pharmacist at every assessment interval. Coding is done according to the Dutch Pharmacotherapeutic Compass 2011 [33].

### Intervention

The intended goal of the intervention is to increase masticatory activity and ultimately, QoL. Therefore, the basic conditions to enable mastication have to be met; one has to have a healthy mouth and must be given food that requires chewing. This results in an intervention aimed at improving oral health care and increasing food consistency.

Oral health care comprises brushing the teeth at least once a day, for at least one minute per jaw. This is a minimum requirement, brushing the teeth twice daily is recommended, in combination with the use of toothpicks and dental floss. Clinical lessons are offered to train the nursing staff in providing this oral health care. Instructions on how to care for a dental prosthesis is also topic of these lessons. The specific skills needed for providing oral health care are also practiced. This is in concordance with the 2007 ‘Guideline oral health care in (residential) care homes for elderly people’ [34].

Increasing food consistency is achieved by: a) evaluation of the need for pureed foods and b) offering food of tougher consistency. Due to apraxia (i.e. inability to perform tasks or movements) or dysphagia (i.e. difficulty in swallowing), some participants are not able to chew and swallow food of normal consistency; hence, their food needs to be pureed. However, some participants might be given pureed foods without medical need, for example out of convenience for the nursing staff. A qualified speech therapist can diagnose swallowing disorders and therefore the need for pureed food. Participants who are given pureed food without medical need will be reintroduced to more solid foods. All participants able to masticate normally are offered food of tougher consistency, such as apples, bread including its crust, crunchy cookies, raw vegetables and salads.

### Outcome variables

#### Quality of Life

The primary outcome variable is QoL, assessed with the Qualidem questionnaire [35]. The Qualidem is considered the preferred instrument [36] for rating QoL for elderly persons with dementia and is appropriate for large and small-scale settings [37]. A proxy is asked to rate observable behaviors on a 4-point Likert scale (i.e. ‘never’, ‘rarely’, ‘sometimes’, and ‘often’). Statements are for example: ‘is cheerful’, ‘refuses food’, ‘smiles’, or ‘wants to stay in bed’. For each statement, 0, 1, 2 or 3 points are awarded. The most positive outcome is given the highest point value (e.g., ‘smiles often’ is given 3 points, ‘refuses food often’ is given 0 points). A higher score suggests a higher QoL. The Qualidem score can be divided into several subscales. Not all subscales are appropriate for participants with severe dementia, so only the four subscales recommended for this group [38] will be included; ‘care relationship’ (0-21 points), ‘positive affect’ (0-18 points), ‘restless tense behavior’ (0-9 points), and ‘social isolation’ (0-9 points). The maximal score is 57 points.

As described earlier, QoL comprises many aspects of life. In order to assess this multicomponent aspect of QoL, the following secondary outcome variables are included.

#### Cognition

Cognition, especially memory and executive function, is investigated using a pen-and-paper-based neuropsychological assessment. Trained examiners, blind for the intervention, will visit the participants at the care unit. First, the participant is screened with the MMSE. Based on the MMSE score, a set of neuropsychological tests will be conducted. If a participant is not able to give a single adequate response on any of the MMSE questions (i.e. MMSE score is zero) no further cognitive testing will take place. Participants scoring 1-4 on the MMSE will take the first four tests described below, and participants scoring 5 or higher on the MMSE will perform these tests and three additional tests (also described below). All these tasks are complementary as they assess different cognitive skills.

#### Category fluency

Category fluency is assessed in two separate instances, by asking the participant to name either as many animals or as many professions as possible in one minute [39,40]. Time is measured with a stopwatch, and all responses are recorded. Identical responses are counted only once (e.g., horse, horse; doctor, doctor), responses assigning gender (e.g., lion, lioness; steward, stewardess) are counted as two correct responses. These rules are explained to the participant before starting. Faulty responses are ignored (i.e. not counted nor subtracted). If the participant starts a random conversation or remains quiet, a gentle reminder (‘can you name any other animals/professions?’) is given. The obtained score is the amount of correct responses.

#### Memory and attention

Memory and attention are assessed by verbally presenting sequences of numbers to the participant, who has to repeat them [41]. The sequences start out with a length of two digits, and after three items, one extra digit is added. The task is cut off when two out of three responses are incorrect. Only correct responses are counted; faulty responses are ignored. The participant is allowed to make corrections. Maximal score is 21.

#### Working memory

Working memory is assessed with a digit span backwards task, which is virtually the same as above; only this time the participant has to give the response in the reverse order [41]. New sequences of digits are used. The task is cut off when two out of three responses are incorrect. Only correct responses are counted; faulty responses are ignored. The participant is allowed to make corrections. Maximal score is 21.

#### Visuospatial function

In order to assess visuospatial function, incomplete line drawings are shown, while the participant has to indicate what the images represent [39]. The drawings are of increasing difficulty, showing animals or everyday items or situations (e.g., a fish, a book, or a man carrying something heavy). After five incorrect responses, the task is cut off. In case of incomplete responses (e.g., ‘a man’ instead of ‘a man carrying something heavy’), the examiner asks the participant to elaborate: ‘please describe everything you see’. Only correct responses are counted; faulty responses are ignored. The participant is allowed to make corrections. Maximal score is 20. If a participant scores 5 points or higher on the MMSE, three tests are added:

#### Verbal long term memory

To assess verbal long term memory, the examiner reads out loud a list of eight everyday words (such as ‘pencil’ or ‘bird’), which the participant must repeat from memory after each presentation; this is repeated five times [42]. Points are awarded for correct responses; the maximal score is 40. After approximately 10 minutes, a delayed free recall and recognition condition will be administered. During the recognition condition, the participant has to indicate whether a word does or does not belong to the original list. Sixteen words are now read out loud, the eight original words and eight new words. Maximal score for free recall is 8, maximal score for recognition is 16.

#### Nonverbal episodic memory

A visual memory task is used to measure nonverbal episodic memory [41]. A card with eight red squares printed on it is placed between the examiner and the participant. The examiner taps the squares in a certain order which the participant is asked to repeat. The sequences start with a length of two squares and after two trials the sequences are lengthened with square. The task is cut off when both responses of a certain length are incorrect. Only correct responses are counted; faulty responses are ignored. The participant is allowed to make corrections. Maximal score is 14.

#### Nonverbal working memory

By using virtually the same task as above, nonverbal working memory is assessed [41]. This time the participant is asked to give the response in the reverse order, the printed squares are colored green and new sequences are used. The task is cut off when both responses of a certain length are incorrect. Only correct responses are counted; faulty responses are ignored. The participant is allowed to make corrections. Maximal score is 14.

#### Assessment of mood

Mood is assessed with two questionnaires regarding observable behaviors, measuring either depression or agitation. Both questionnaires will be filled out by a proxy, typically a member of the permanent nursing staff.

#### Depression

The presence or absence of depression is qualified using the Cornell Scale for Depression in Dementia (CSDD; [43] Dutch version (CSDD-D; [44]. Nineteen statements about the participant are scored on a Likert scale: a = not able to judge; 0 = absent; 1 = slightly or variably present; and 2 = severe. Maximal score is 38 points; a higher score indicates the presence of more depressed behaviors. Statements refer to behaviors such as suicidal tendencies and facial expressions of sadness or fear, but also to weight loss or sleep disturbances. A score below 8 is considered to be within the normal range and a score of 8 and higher is indicative of depression [43,45].

#### Agitation

The amount of agitated behaviors is scored using the Dutch version of the Cohen-Mansfield Agitation Inventory (CMAI) [46]. A total of 29 observable behaviors are scored on a 7-point Likert scale. Items are behaviors such as spitting, (verbal) aggression, biting, screaming, complaining, and others. The behaviors can be observed never (1), less than once a week (2), once or twice a month (3), several times per week (4), once or twice per day (5), several times per day (6), or several times per hour (7). Minimal score is 29; maximal score is 203. A higher score is indicative of more agitated behaviors.

#### Assessment of independence

The ability to perform activities of daily living is assessed with the Katz index of Independence in Activities of Daily Living [47]. This index rates five activities and the ability to perform them unaided. They are: dressing, using the toilet, eating, moving around, and taking a shower or bath. A sixth question regards whether the participant is incontinent. A score of 1 indicates independence, score 2 stands for ‘needs some help’, and a score of 3 is given when someone is completely dependent (or, completely incontinent). Minimal score is 6; maximal score is 18.

#### Assessment of the rest-activity rhythm

The rest-activity rhythm is a circadian rhythm that reflects the sleep-wake rhythm in an indirect way. The rest-activity rhythm is measured during a week (7*24 hours), using an Actiwatch activity monitor (Cambridge Neurotechnology Ltd., Cambridge, United Kingdom). The Actiwatch is a small device that is worn on the wrist. It is placed on the dominant arm, unless this leads to agitation (e.g., due to presence of a wristwatch or bracelet). The Actiwatch is taken off only during showering since it is not waterproof. The Actiwatch records the motions of the arm, which are a proxy for overall activity [48]. The recorded data are analyzed for the following variables:

#### Interdaily stability (IS)

The interdaily stability is a measure for the degree of similarity of activity patterns within the measured period. A stable rhythm is characterized by a higher IS score; scores are between zero and 1.

#### Intradaily variability (IV)

The intradaily variability is the difference in activity levels in periods throughout the day. A normal activity pattern shows low IV; a sinusoid pattern results in a score of zero, a score of 2 is obtained for noise, and higher scores can arise obtained due to peaks in activity.

#### Relative amplitude (RA)

The relative amplitude is calculated by dividing the difference between the means of the ten most active hours (M10) and the five least active hours (L5) by the sum of these means within a day/night cycle.

$$\mathrm{RA}=\frac{M10-L5}{M10+L5}$$

A larger RA is associated with a more pronounced wake/sleep cycle. A more detailed description of these variables and their analysis is available elsewhere [48].

#### Assessment of blood pressure

Blood pressure is measured in millimeters of mercury (mmHg) with a blood pressure monitoring device (SpaceLabs Medical Inc., Redmond, Washington, United States of America). The participant is sitting down quietly, unless they are bedridden in which case they are lying down. Systolic blood pressure and diastolic blood pressure are noted, as well as heart rate. Hypertension is defined as systolic blood pressure higher than 160 mmHg and/or diastolic blood pressure over 95 mmHg [49].

#### Assessment of masticatory function

Mastication can be assessed in several ways. Most notably is the distinction between subjective, self-rated masticatory ability and the objective outcome masticatory efficiency (also described as masticatory performance) [50,51]. In the present study, masticatory ability is not assessed, since the cognitively impaired participants are not likely to be able to reliably answer questions about their chewing capacity. Participants’ masticatory efficiency is assessed using several techniques. First of all, the dental status is recorded: is the participant dentate, is he/she wearing a partial of full prosthesis, etc. If possible, the dental records are used for obtaining this information, otherwise, nursing staff is interviewed, or a visual inspection is performed. Secondly, several assessments are taken:

#### Occluding units

The number of pairs of upper and lower teeth that touch each other when the mouth is closed (i.e. occluding units) are measured using dental modeling wax (Alminax; Müller & Weygandt, Büdingen, Germany). The wax is solid at room temperature and becomes soft when immersed in warm water. The participant bites down on a plate of softened wax and then it is allowed to harden again. Determination of the number of occluding units is done by visual inspection. In complete dentitions, 8 teeth are present in each quadrant (two incisors, one canine, two premolars, and three molars). Thus, the maximal score is 16 occluding units (i.e. 32 teeth).

#### Maximal mandibular excursions

Active, voluntary, mandibular mobility is assessed as the distance between the upper and lower incisal edges, in millimeters, and is measured with a flexible plastic ruler (Dental Union, Nieuwegein, The Netherlands) [30,52]. First, the participant is asked to open his/her mouth as wide as possible without experiencing any pain. Next, the participant is encouraged to open his/her mouth as far as possible, even if this is painful, thus enabling the assessment of the maximal mouth opening. During the maximal opening, the participant is continuously encouraged. Maximal protrusion (i.e. outward forward movement of the mandible) is assessed by asking the participant to push the lower jaw forward as far as possible. Laterotrusion (i.e. outward movement of the jaw to the side) to the left and right are also measured. After every mandibular excursion, the participant is asked whether this is painful and if so, where the pain is located and what the intensity of the pain is. Pain location is recorded as joint area, pre-auricular, cheek area, floor of the mouth, temporal area, and other. Pain intensity is recorded on a 5-point Likert scale: 0 = no pain, 1 = tenderness, 2 = mild pain, 3 = moderate pain, and 4 = severe pain. Overjet (i.e. the antero-posterior distance between the upper and lower incisors), overbite (i.e. the vertical overlap of the upper and lower incisors), and midline deviation (i.e. the horizontal distance between the upper and lower dental arch midlines) are recorded while the participant rests in occlusion. The vertical maximal opening is corrected for overbite, the protrusion is corrected for the overjet, and the laterotrusions are corrected for the midline deviation.

#### Maximal voluntary bite force (MVBF)

The maximal voluntary bite force (MVBF) is measured with the VU University Bite Force Gauge (VU-BFG). The VU-BFG is a hand-held device which uses a load cell to measure maximal voluntary bite force in kilograms. The VU-BFG can be used centrally between the incisors or unilaterally between the (pre-) molars. For this study, the VU-BFG will be used between the incisors. The participant is instructed to bite as long and hard as possible and is encouraged continuously during the sampling. If the sampling fails (e.g., due to losing the prosthesis), a second attempt will be made after a rest period. Maximal sampling time is 20 seconds, and sampling takes place at a frequency of 50 Hz. All bite force samples are logged; the highest (i.e. peak) value is used as MVBF.

#### Mixing ability

In order to quantify actual masticatory performance, a mixing ability test in which the participant has to orally knead two viscoelastic colored materials [53,54] is used. A four-gram sample made of blue and pink chewing gum (Bubblicious® Ultimate Original and Twisted Tornado; Cadbury, London, United Kingdom) is given to the participant, with the instruction to chew naturally. The sample resembles a piece candy due to its general appearance (bicolor capsule-shaped sample in a cellophane wrapper) and smell (sugary and sweet) which makes it easier for the participant to accept it as test food. A casting mold ensures consistent production of samples. Several protocols use a fixed number of chewing cycles, e.g. [53]. However, participants suffering from dementia may find it hard to count and chew at the same time. An observer cannot accurately distinguish individual chewing cycles either, due to for example swallowing in between chewing motions, movements of the head while chewing (e.g., looking around), presence of a tremor, or obscuring of the jaw due to facial features such as sagging skin or beard (pilot data, not shown). Using a fixed amount of chewing cycles is therefore not possible in this population. Since in healthy people the average chewing frequency is stable at approximately 1.4 hertz (1.45 Hz for women and 1.30 Hz for men; [55], the assumption is made that this is also the case in persons suffering from dementia. This was confirmed in a pilot study (data not shown) and therefore a standard chewing time of 20 seconds will be used. A stopwatch is used to measure time. After digital optical analysis, a mixing ability score is obtained [53,54].

### Statistical analysis

The data from this prospective longitudinal matched cluster randomized single-blind multicenter study will be analysed using a linear mixed model. The fixed effects are *‘condition’* and *‘time’*; the linear ‘*condition x time’* interaction is the effect parameter of interest and *‘care unit’* and *‘participant (within the care unit)’* are the random effects (both intercepts and slopes). An intention to treat analysis will be performed.

The baseline (cross-sectional) data will also be analysed for Pearson’s and/or Spearman’s coefficients, to establish correlations between the primary outcome variable QoL and the secondary outcome variables (viz., cognition, mood, independence, rest-activity rhythm, blood pressure, and masticatory function). Additionally, linear regression analyses will be performed to study the independent contribution of the various secondary outcome variables to QoL.

A baseline comparison will be performed to make sure that the intervention group and control group are similar with regards to variables that are not of primary interest (i.e. the covariates and confounders) and the outcome variables. Any difference between the groups afterwards is then likely due to the intervention. If there are differences between the groups prior to the intervention, the baseline scores will be incorporated as covariates in the analysis.

### Ethical considerations

This study is approved by the Medical Ethical Committee of the VU University Medical Centre (METc VUmc; ref: **2010–342**) according to the Declaration of Helsinki (2008). The research has been included in the general assessment and registration form (ABR form) (ref: NL33230.029.10) and in the Netherlands National Trial Register (NTR) (ref: NTR1561).

## Discussion

The described study is a prospective longitudinal matched cluster randomized single-blind multicenter study, investigating the effect of an intervention aimed at increasing masticatory activity by increasing and improving oral health care and the consistency of the diet. Participants in this study are elderly people suffering from dementia and receiving institutionalized care. The intervention is performed by daily nursing staff.

Strengths of this design are the direct clinical applicability of results; a transfer to the clinic does not need to be made since all work is done in a clinical setting. The intervention is performed by the nursing staff, allowing immediate assimilation into daily care. By taking baseline measurements and also repeated follow-up assessments, a thorough insight into the effect of the intervention is obtained. Chance findings are less likely to occur, and the effect over time can be made clear. The broad spectrum of data collection allows for a further widening of the research scope. Besides QoL, important variables such as cognition, mood, independence, and the rest-activity rhythm are assessed. Also, the research methods are varied; both qualitative and quantitative measures are used. By combining these two research methods, we obtain both quantitative, objective data about ‘how’ the intervention is effecting the outcome variables and qualitative, more subjective data, which is descriptive and can provide insight into the ‘why’ an intervention does or does not have an effect. Quantitative data can provide insight in effect sizes and underlying mechanisms, is not subject to interpretation and not dependent on a proxy’s mood and cognition (e.g., [13]). Nonparametric testing, used for qualitative data, is stricter and therefore if results are found, they tend to be more robust. The heterogeneity of the research population is (partly) eliminated by matching and cluster randomizing the care units.

There are of course also some aspects of the study design that might prove to be shortcomings. The Consolidated Standards of Reporting Trials (CONSORT) guidelines [56] include the practice of blinding of participants, the nursing staff, and the assessors. However, since the nursing staff in this protocol plays a vital role in providing the altered care as the intervention agents, they cannot be blinded. A placebo treatment which resembles oral health care in such a way that the nursing staff would not notice the difference without actually being effective is, to our knowledge, not available. The same holds true for the participants; there is no placebo treatment available and therefore, participants in the control group receive ´care as usual´. This ´care as usual´ for dependent elderly could, and one might even argue, should, include oral health care, but it has been shown that this is often unfortunately not the case (e.g., [26,28,57-60]). In contrast to the nursing staff and the participants, the trained examiners are blinded for the participants’ allocation in either the intervention or control group.

There will also be a certain amount of variety in the nature of interventions due to local differences in nursing homes. For example, the presence of a dentist or dental hygienist and/or the possibility to cook rather than order meals, will shape the specific details of the local intervention. This flexibility might be perceived as a weakness in the study design, because the intervention is not uniform across the several care facilities; however, a practical implementation of any findings would be subject to the same factors, making the study design clinically relevant. Furthermore, the use of matched cluster randomization, with one care organization always providing both an intervention and control group, is a good way to control for this variation. In the CONSORT guidelines, this design is described as stratification by center and is considered appropriate to control for differences such as can occur in multicenter studies [56].

Effects in the increased food consistency group cannot be attributed to mastication per se, but can also be considered as an effect of the changed diet, enhancing for example, vitamin intake. Similarly, any effects of oral health care cannot purely be attributed to increased mastication; for example lowered incidence of oral pain or inflammation might also have effects on QoL. Despite these limitations, we argue that the outcomes of this study will point the direction for further research into these areas and will provide important insights in the topic of gerodontology in particular and dementia care in general.

## Abbreviations

AD: Alzheimer’s disease; ADL: Activities of daily living; ApoE: Apolipoprotein E; CMAI: Cohen-mansfield agitation inventory; CONSORT: Consolidated standards of reporting trials; CSDD-D: Cornell scale for depression in dementia; DLB: Dementia with Lewy bodies; FTD: Frontotemporal dementia; IS: Interdaily stability; IV: Intradaily variability; L5: 5 Least active hours; M10 10: Most active hours; mmHg: Millimeters of mercury; MMSE: Mini mental state examination; MVBF: Maximal voluntary bite force; QoL: Quality of life; RA: Relative amplitude; RAI: Resident assessment instrument; SES: Socioeconomic status; VaD: Vascular dementia; VU-BFG: VU University bite force gauge.

## Competing interests

The authors declare that they have no competing interest.

## Authors’ contributions

RAFW performs the study and drafted the manuscript. FL participated in the design and coordination of the study and critically revised the manuscript. DLK provided the statistical analysis and power calculations. JT is second study performer and critically revised the manuscript. EJAS devised the study, participated in the design and coordination of the study and assisted in writing the manuscript. All authors read and approved the final manuscript.

## Pre-publication history

The pre-publication history for this paper can be accessed here:

http://www.biomedcentral.com/1471-2377/13/26/prepub
